# Host Associations of *Culex* (*Melanoconion*) *atratus* (Diptera: Culicidae) and *Culex* (*Melanoconion*) *pilosus* from Florida, USA

**DOI:** 10.3390/insects10080239

**Published:** 2019-08-03

**Authors:** Lawrence E. Reeves, Isaiah Hoyer, Carolina Acevedo, Nathan D. Burkett-Cadena

**Affiliations:** Florida Medical Entomology Laboratory, Entomology and Nematology Department, University of Florida, 200 9th St. SE, Vero Beach, FL 32962, USA

**Keywords:** *Melanoconion*, *Anolis*, *Culex*, mosquito, blood meal analysis, Florida

## Abstract

Characterizing the host-use patterns of mosquitoes is an essential component of understanding the transmission dynamics of mosquito-vectored pathogens. The host associations of two species of the medically important *Culex* subgenus *Melanoconion*, *Culex atratus,* and *Culex pilosus* are unknown or unclear, respectively. Both species have wide neotropical distributions. In the United States of America (USA), *Culex pilosus* occurs throughout the southeastern coastal plain, while *Culex atratus* is restricted to the southern Florida Peninsula. Using PCR-based blood meal analysis, we investigated the host associations of *Culex atratus* and *Culex pilosus* that were collected from Everglades National Park, Florida, USA We identified the host species of 208 *Culex atratus* and 168 *Culex pilosus*. Both species were narrowly associated with reptilian host species, particularly native and non-native lizards of the genus *Anolis*. Sampled *Culex atratus* exclusively fed on reptilian hosts, with >99% of blood meals derived from *Anolis* lizards. *Culex pilosus* fed predominantly from reptiles, but avian and mammalian blood meals were also detected. Of these, 92% of blood meals were derived from *Anolis* species. For both species, *Anolis sagrei*, an invasive exotic lizard in Florida, was the most frequently detected host species. These data indicate that *Culex atratus* and *Culex pilosus* are specialists of reptilian hosts, particularly *Anolis* lizards.

## 1. Introduction

The mosquito species that comprise the *Culex* subgenus *Melanoconion* are diverse, distributed throughout the Americas, from Canada to Argentina, and are difficult to morphologically distinguish [1]. Of the 160 described *Melanoconion* species, most are Neotropical in distribution with a center of diversity in the Amazonian Region of northern South America [1]. Ten species of *Melanoconion* occur in the continental United States of America (USA) and eight of these are present in Florida [2]. Adults and larvae of one, *Culex panocossa*, were recently collected in southernmost peninsular Florida, and likely represent recent introduction and establishment [3]. Several *Melanoconion* species are vectors or putative vectors of pathogenic arboviruses. *Culex erraticus* and *Culex taeniopus,* for example, transmit eastern equine encephalitis virus [4] and Madariaga virus [5], respectively, while *Culex cedecei*, *Culex iolambdis*, *Culex taeniopus,* and others are enzootic vectors of various Venezuelan equine encephalitis (VEE) virus subtypes [3,6,7]. Aside from the known or suspected pathogen vectors, the biology and ecology of the majority of *Melanoconion* species have not been well characterized. We investigated the host associations of two *Melanoconion* species in southern Florida, *Culex atratus* Theobald and *Culex pilosus* (Dyar & Knab), both of the Melanoconion Section, Atratus Group and Pilosus Group, respectively [8]. 

*Culex atratus* and *Cx. pilosus* are widely distributed in the American tropics, both being found throughout much of South America [9], the Caribbean [10], and southern North America [2,3]. *Culex atratus* is found throughout northern South America, the Caribbean, and southern Florida, but is not known from Central America, apart from Panama [1,2,11]. *Culex pilosus* occurs in several states of the southeastern USA, throughout Central America, and as far south as central Argentina [1,12,13]. Few data are available on the host groups fed upon by these mosquitoes despite the very wide distributions of these two mosquito species, and the general importance of *Melanoconion* species in the transmission of zoonotic arboviruses [11]. Without host associations, it is difficult for arbovirus researchers and public health professionals to “incriminate” or “exonerate” potential vectors species in the transmission of zoonotic pathogens.

Host associations of *Cx. atratus* are currently unknown. While some early studies authors [14,15] reported *Cx. atratus* to be a major biting nuisance to humans, Page found that few *Cx. atratus* females were attracted to human bait [16]. *Culex atratus* were collected with donkey-baited traps in Jamaica [10], although whether or not donkeys were actually bitten was not reported. In the 1970s, Edman [17] investigated the host associations of *Melanoconion* mosquitoes from sites in Florida, USA. Notably, *Cx. atratus* was absent from Edman’s collections in Everglades National Park, where *Cx. atratus* has been known to occur since the 1960s [18,19]. In Florida, USA, *Cx. atratus* is represented by two forms, *Culex atratus sensu stricto* and *Culex atratus* B, which are genetically distinct, yet indistinguishable by external morphology [20,21]. The distributions of these two forms and potential differences in their ecologies, which include host associations, are not known. 

Host associations of *Cx. pilosus* are also unclear, based upon the limited work that has been performed. Edman [17] concluded that *Cx. pilosus* predominantly feeds on reptiles in Florida. However, in Louisiana Cupp and Stokes [22] captured female *Cx. pilosus* while using dog-baited traps and identified 73 out of 102 (71.2%) *Cx. pilosus* blood meals, of which, none were derived from reptilian hosts. From these, birds (26.0%), humans (20.5%), horses (32.9%), and dogs (20.5%) were identified as hosts. In a recent study, Reeves et al. [23] used DNA barcoding to identify five *Cx. pilosus* blood meals from northern Florida and found four were derived from reptilian hosts and one from an avian host. Otherwise, all previous studies investigating the host-use of *Cx. pilosus* relied on the serological methods of host identification that made species-level host identification difficult. 

Subgenus *Melanoconion* is a medically important group of mosquitoes, containing species that are important to the transmission of zoonotic arboviruses. The host associations of two species from Florida, *Cx. atratus* and *Cx. pilosus*, are not well understood. There are no published blood meal analysis data for *Cx. atratus*, and previous work reports contrasting patterns of host use for *Cx. pilosus*. We collected the females of *Cx. atratus* and *Cx. pilosus* from Everglades National Park and surrounding areas in southern Florida and used PCR-based blood meal analysis to investigate and quantify the host use of these two mosquito species.

## 2. Materials and Methods 

### 2.1. Study Sites and Sampling Protocol

**Figure 1:**
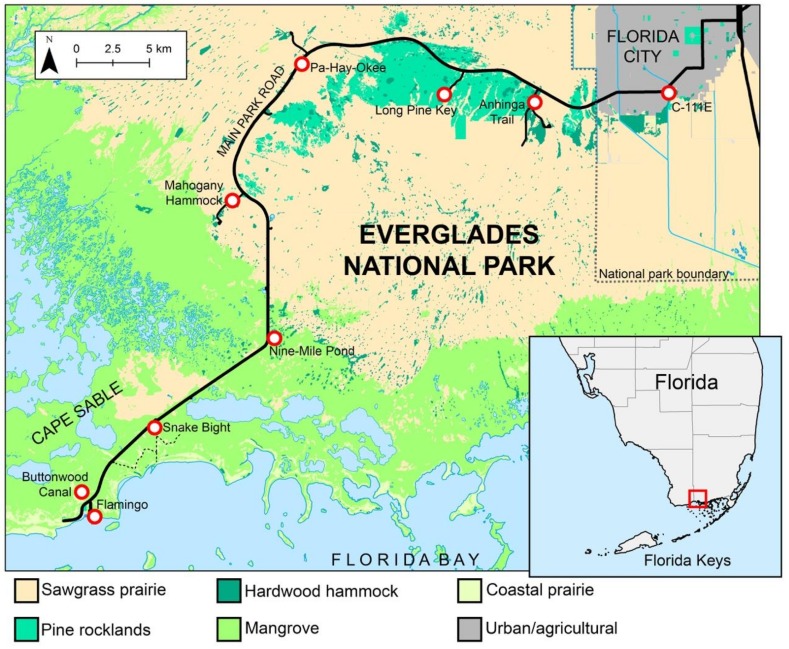
Map of sampling area indicating the sites where blood fed female *Culex atratus* and *Culex pilosus* were collected (red circles). The red box in the inset map of the southern Florida Peninsula indicates the figured area.

Mosquitoes were sampled at eight sites in Everglades National Park, Florida, USA and one external site while using resting shelters [24,25] and aspiration of natural resting sites (Table 1). All of the sites were located along State Highway 9336/Main Park Road, between Homestead, Florida, USA and Flamingo, Florida, USA (Figure 1). The external site, the C-111E Canal, was located in a tropical hardwood hammock, which was approximately 5.1 km from the national park border. Mosquito samples were collected from Everglades National Park between October 2015 and June 2016, and between February 2017 and July 2017 under permit numbers EVER-2015-SCI-0054 and EVER-2017-SCI-0011. The mosquitoes were collected during sampling events lasting two to five consecutive days, every other month. The numbers and placement of resting shelters varied between sampling events. In general, 10–16 shelters (one to two per site) were placed in the field at the beginning of each sampling event and removed at the end of the event. 

Mosquitoes were removed from the resting shelters each morning of the sampling event between 0900 and 1100 h while using a battery-powered aspirator constructed from a Dustbuster™ (Black & Decker^®^, Towson, MD, USA) handheld vacuum modified to hold plastic collection canisters with wire mesh bottoms (BioQuip Products, Rancho Dominguez, CA, USA), as described in Bingham et al. [26]. Various methods were used to minimize the escape of resting mosquitoes during collection including using baffles [23,24]. The same aspirators were used to collect mosquitoes from natural resting sites (e.g., root masses of fallen trees, bases of tree trunk, tree cavities, dense vegetation). The aspirator, powered on, was swept through natural resting sites at 5–10 min. intervals. The use of this method varied between sites and sampling events due to the availability and accessibility of natural resting sites.

Immediately following collection, mosquitoes in collection canisters were either transported alive on ice inside a cooler to Flamingo (at the southern terminus of State Highway 9336/Main Park Road), or maintained on dry ice for two to three days until transportation to the Florida Medical Entomology Laboratory, Vero Beach, Florida, USA. At Flamingo, the mosquitoes were killed by exposure to ethyl acetate-soaked plaster for ~10 min. and examined under stereoscope magnification. Any female mosquito with visible evidence of blood in its abdomen was separated, being identified to species level [12], and preserved on Whatman^®^ four-sample Flinders Technology Associates (FTA) Classic Cards [27]. Each mosquito was assigned a unique identifying number and transferred to the sampling area of the FTA card. The apical end of a sterile pipette tip was placed between the thorax and the abdomen of the mosquito, and pressure was gently applied to release the blood meal onto the card. The blood meal was spread around the sampling area of the card until all of the viscous droplets were absorbed. The preserved blood meals were air-dried for ~10 min. and stored in plastic bags until DNA extraction. Mosquitoes transported to the Florida Medical Entomology Laboratory were maintained frozen (−20 °C) until morphological identification while using a chill table.

### 2.2. Blood Meal Analysis

DNA was directly extracted from blood meals preserved on FTA cards while using the hot sodium hydroxide and tris (HotSHOT) method [28] or from whole mosquito specimens. The DNA extraction protocol for FTA card-preserved mosquito blood meals, followed a previously published protocol [27]. Two 1 mm sections of each blood meal were removed from the card with a 1 mm hole punch. The punches were transferred to the same 0.2 mL PCR tube or 0.2 mL well of a 96-well PCR plate while using flame-sterilized forceps and 25 µL of lysis solution (25 mM NaOH and 0.2 mM EDTA) were added. The tubes/plate were sealed and incubated at 95 °C for 30 min., followed by 4 °C for five min. in a BioRad DNA Engine thermocycler. Following incubation, 25 µL of neutralization solution (40 mM Tris-HCl) were added to each tube/well. The samples were briefly vortexed and stored at −20 °C until PCR. DNA was extracted from whole mosquito specimens using Instagene Matrix or Chelex resin (BioRad, Hercules, CA, USA) while using published protocols [29,30,31].

Extracted DNA was used as DNA template in PCRs that were intended to amplify regions of the vertebrate cytochrome *c* oxidase subunit I (COI) gene or 16S ribosomal RNA gene while using four primer pairs (Table 2). Host identifications using the vertebrate COI gene used an unstructured hierarchical approach and the primer sets described in Reeves et al. [32]. Briefly, DNA extracted from mosquito blood meals were used as templates in PCR reactions while using one of three primer sets: VertCOI_7194_F and Mod_RepCOI_R (expected amplicon length 395 bp), Mod_RepCOI_F and VertCOI_7216_R (244 bp), and Mod_RepCOI_F and Mod_RepCOI_R (664 bp). The products of successful amplification reactions were sequenced. DNA templates that failed to amplify were subsequently used in a second reaction with a different primer set, and products were sequenced if amplification was successful. If amplification failed, the template was used in a third reaction, while using the remaining primer set. If the third reaction did not produce an amplicon, no further steps were taken, and the blood meal was not identified. 

For all reactions intended to amplify vertebrate COI templates, other than the primer sequence, the thermocycling conditions, reagents, and reagent concentrations were identical for all amplification reactions. Each reaction followed the protocol of Reeves et al. [32] and each was performed in a total volume of 20 μL that consisted of 10 μL of 2.0X ApexTM Taq RED Master Mix^®^ (Genesee Scientific, San Diego, CA, USA), 0.75 μL forward primer (10 μM), 0.75 μL reverse primer (10 μM), 7.5 μL sterile, double-distilled water, and 1 μL of extracted DNA. Thermocycling conditions followed a standard profile for all reactions that consisted of an initial denaturing step of 94°C for 3 min., followed by 40 cycles of 94 °C for 40 s, 48.5 °C for 30 s, and 72 °C for 60 s, and a final extension step of 72 °C for 7 min.

Reactions that were intended to amplify vertebrate 16s templates followed previously published protocols [29,30,31] and were performed in a total volume of 25 µL that consisted of 0.625 μL of forward primer 16L1 (20 μM), 0.625 μL of reverse primer H3056 (20 μM), 2.5 μL 10× PCR buffer, 1.5 μL MgCl_2_ (50 mM), 2.5 μL dNTP mix (2 mM each), 0.5 μL Taq DNA polymerase, 2.5 µL DNA template, and 14.25 μL molecular grade water. Thermocycling conditions for all reactions targeting 16s templates consisted of 4 min. at 95 °C, 35 cycles of 95 °C for 30 s, 62.5 °C for 30 s, 72 °C for 30 s and a final extensions step of 72 °C for 7 min.

Negative controls in which sterile double-distilled water replaced extracted DNA were included in all of the reactions to monitor for contamination. The reaction products were stained with ethidium bromide, electrophoresed on a 1.5% agarose gel for approximately 30 min., and visualized under ultra-violet light. A 50 bp DNA ladder was electrophoresed alongside PCR products to estimate the size of any visible amplicons. Products from all reactions that showed the presence of an amplicon of the expected size were sent to Genewiz^®^ (South Plainfield, NJ, USA) or Eurofins^®^ (Louisville, KY, USA) for purification and DNA sequencing via the Sanger method [33].

Sequence files from positive reactions were checked and edited for quality while using the bioinformatics software Geneious version R10 [34]. Sequences were then submitted to the Barcode of Life Datasystems [35] sequence database for identification. Sequences that were >98% similar to referenced sequences, or independently obtained reference sequences [23,32] were identified as the corresponding species.

### 2.3. Statistical Analysis

Fisher’s exact test [36] was used to test for differences in the effect of primer pair on blood meal results. Separate analyses were performed for *Cx. atratus* and *Cx. pilosus*, while using the count of blood meals attributed to each host class or that did not amplify as the dependent variable. Fisher’s exact test was selected to assess whether the relative proportions of blood meal analysis results were similar across the primer sets, because the expected numbers for some categories were small.

## 3. Results

We collected and analyzed blood meals from a total of 260 *Cx. atratus* and 248 *Cx. pilosus* (Table 3). Blood meal analysis successfully identified a host species for 208 (80%) *Cx. atratus* and 168 (67.7%) *Cx. pilosus*. Amplification was successful for 81.4% of *Cx. atratus* and 67.7% of *Cx. pilosus* samples attempted. One *Cx. pilosus* blood meal was derived from more than one host species. The blood meal analysis results across primer sets and the distribution of host class counts for each primer set were relatively even. Fisher’s exact test indicated that there were no differences in the effect of primer set on blood meal analysis results for *Culex atratus* (*p* = 0.446) and *Culex pilosus* (*p* = 0.79).

**Figure 2:**
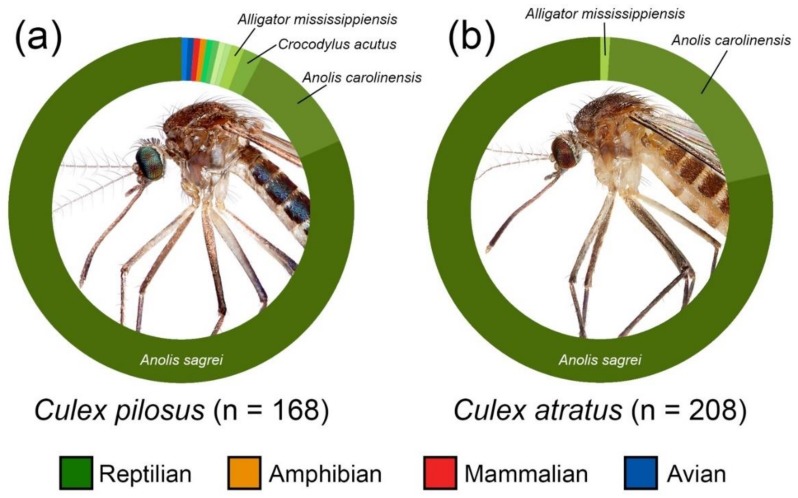
Composition of hosts identified from samples of (**a**) *Culex pilosus* and (**b**) *Culex atratus* blood meals collected in Everglades National Park, Florida USA by host class. Colors (green, orange, red, blue) indicate host class. Shades of each color indicate host species within each respective class. Host species represented by >1 blood meal are labeled.

From the 208 *Cx. atratus* blood meals that were successfully identified, just three species of vertebrate hosts were detected. Two lizard species of the genus *Anolis*, *Anolis sagrei* (brown anole) and *Anolis carolinensis* (green anole) were the most frequently detected hosts, together representing 99% of identified *Cx. atratus* blood meals (Figure 2). Of these, 163 blood meals (78.4%) were attributed to *A. sagrei*, and 43 (20.7%) were attributed to *A. carolinensis*. Only two *Cx. atratus* blood meals were identified from other host species, both from *Alligator mississippiensis* (American alligator). Fifty-two *Cx. atratus* blood meals were not amplified by PCR.

The host-use patterns of *Cx. pilosus* were similar to those of *Cx. atratus,* but with greater host breadth (Figure 1). *Anolis* lizards were the most frequently detected hosts (92.2%, together). In addition to *A. sagrei* and *A. carolinensis*, one blood meal, from the C-111E Canal site, was derived from *Anolis equestris* (knight anole). *Anolis sagrei* and *A. carolinensis* were the first and second most frequently identified host species, representing 81.5% and 10.7% of all identified blood meals, respectively. Altogether, twelve host species were detected from the sample of 168 identified *Cx. pilosus* blood meals. Nine other vertebrate host species were detected in addition to the three *Anolis* species, each with <3 detections. These species were primarily reptiles, including species of snakes, turtles, and crocodilians, but three were endothermic hosts: *Cardinalis cardinalis* (northern cardinal), *Nyctanassa violacea* (yellow-crowned night heron), and *Homo sapiens*.

## 4. Discussion

**Figure 3:**
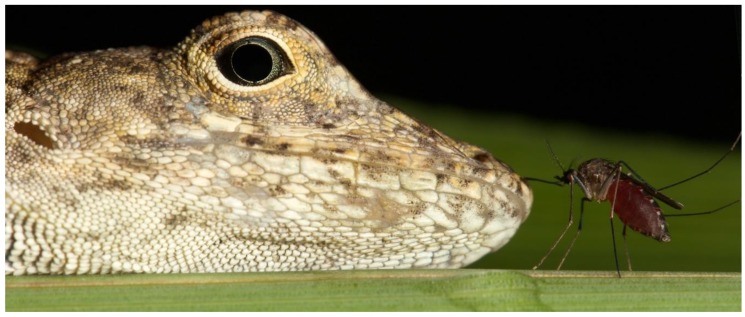
Female *Culex pilosus* feeding from *Anolis sagrei* at Blue Cypress Lake, Indian River County, Florida, USA, 8 July 2018.

The blood meal analysis results of the current study indicated that *Cx. atratus* and *Cx. pilosus* are strongly associated with reptilian hosts, particularly lizards of the genus *Anolis*, at Everglades National Park (Figure 3). This provides the first documented host associations of *Cx. atratus* by blood meal analysis, which suggested that previous indications of *Cx. atratus* posing a biting nuisance to humans [14,15] could be due to misidentifications. These results also provide support for the conclusions of Edman [17] and Reeves et al. [23] that *Cx. pilosus* predominantly feeds on reptiles. 

For *Cx. atratus* and *Cx. pilosus*, 20% and 32% of blood meals, respectively, did not to produce a PCR amplicon. We expect this is due to mosquito digestion-associated degradation of template host DNA. Previous investigations have found that host DNA becomes undetectable by PCR after 36–72 h post-feeding [27]. Following collection of specimens, all of the mosquitoes that contained a visible trace of a blood meal were included in the sample, and it is likely that a portion of the collected blood meals were beyond this host DNA detection timeframe. It is also possible that this timeframe varies between host classes. The red blood cells of mammals are anucleated and they do not contain mitochondria, while those of amphibians, birds, and at least some reptiles are nucleated and contain mitochondria [37]. Our molecular analyses targeted mitochondrial genes that, as a result, are likely more abundant in non-mammalian blood. The impact of this on blood meal analyses that target mitochondrial DNA is unknown, but previous research using the same primer sets effectively identified the mammalian hosts from mosquito blood meals [29,30,31,32].

We found *Cx. atratus* to be abundant in sites throughout Everglades National Park (Table 1, [25]). We observed that mosquitoes were generally less abundant in resting shelter and aspirator collections when the environmental conditions were dry or windy, or when temperatures were low. *Cx. atratus* and *Cx. pilosus* were both common in most sampled habitats (Table 1), but generally more mosquitoes were collected from more complex habitats with increased tree cover (e.g., tropical hardwood hammocks, pine rocklands, and to a lesser extent, mangroves). Future work should explore the environmental and climatic factors that influence activity, abundance, and host associations of these mosquitoes over time in southern Florida. Our finding that *Cx. atratus* is abundant in Everglades National Park conflicts with the results of Edman [17], despite the use of similar collection methods (resting shelters and aspiration of natural resting sites). The discrepancy in abundance between our current work and that of Edman [17], in which *Cx. atratus* was not encountered, might be due to the introduction, establishment and dispersal of (1) an important host (*A. sagrei*); or, (2) an exotic cryptic species of *Cx. atratus*. 

*Culex atratus* blood meals were derived from an especially narrow range of host animals, with 99% of all identified blood meals being obtained from two *Anolis* species. Of these, 79% of blood meals were from just one species, *A. sagrei*. *Anolis sagrei* is now one of the most abundant reptile species in Florida, occurring in every county of the peninsula [38]. It is possible that the establishment of *Anolis sagrei* in Florida enabled *Cx. atratus* to expand its distribution and increase its abundance in the southern half of the state. The exact timing of the establishment and expansion of *A. sagrei* in the Everglades is difficult to determine, but, by 1980, *A. sagrei* was well established in urban areas of the Florida Peninsula [38], subsequent to the work of Edman [17]. *Anolis sagrei* is native to Cuba and the Bahamas and it has been widely introduced outside its native range [39]. 

Florida populations of *Cx. atratus* represent two putative cryptic species, *Culex atratus s.s.* and an undescribed species, *Culex atratus* B [20]. The external morphologies of these putative species are identical, but they are distinguishable by DNA sequence variation and the morphology of the cibarial armature [20]. We dissected and prepared the heads and cibarial armature of a subset of *Culex atratus sensu latu* collected at Everglades National Park during the sampling period. These individuals were consistently identified as *Cx. atratus* B. Further work is required for understanding the distribution of *Cx. atratus s.s.* and *Cx. atratus* B in Florida, and to identify the differences in host associations, if any. It is possible that the distribution of *Cx. atratus* B has expanded northward, as suggested by recent new county records from Vero Beach, Indian River County [29].

If the availability of certain primary hosts is a prerequisite for *Cx. atratus* and *Cx. pilosus*, the presence of *Anolis* lizards may be a factor in their geographic occurrence. The USA distribution of *Cx. atratus* is entirely sympatric with both *A. sagrei* and *A. carolinensis*, and the collection records suggest that it is limited to coastal regions of the southern Florida Peninsula, as far north as Indian River [29] and Manatee County [40]. Like *Cx. atratus*, the primary hosts of *Cx. pilosus* were *A. sagrei* and *A. carolinensis*. The USA distribution of *Cx. pilosus* extends beyond that of *A. sagrei*, and it loosely parallels the distribution of *A. carolinensis* [2,41]. The host associations of *Cx. pilosus* differed from those of *Cx. atratus* in that ~10% of *Cx. pilosus* blood meals were derived from non-*Anolis* hosts. These hosts were largely reptilian, but avian and mammalian hosts were also detected. In comparison, <1% of *Cx. atratus* blood meals were derived from non-*Anolis* host animals. The determinants of geographic distribution for these mosquitoes are unclear, but a greater flexibility of host associations might enable *Cx. pilosus* to occupy a wider range in the USA Similarly, the spread of non-native species, such as *A. sagrei*, which are primary hosts for mosquito species may influence the geographic distribution of mosquitoes and enable them to expand their range.

Overall, and at each site, *A. sagrei* was fed upon more frequently than *A. carolinensis* by both mosquito species. This result may reflect the abundance patterns of the two *Anolis* species. Alternatively, host-seeking and foraging behavior by the mosquitoes may influence the strength of the host associations of these mosquitoes. *Anolis sagrei* and *A. carolinensis* occupy similar ecological niches, and one result of interspecific competition between these species in Florida is niche partitioning [42]. In habitats that are occupied by both *A. sagrei* and *A. carolinensis*, the species are vertically stratified with *A. sagrei* occupying ground and understory microhabitats, and *A. carolinensis* shifting to the canopy and higher microhabitats [43,44]. If female *Cx. atratus* and *Cx. pilosus* concentrate their host-seeking activity to the lower levels of their habitats, they may be more likely to encounter *A. sagrei* rather than *A. carolinensis*, which potentially leads to the host association patterns that we observed. 

In general, the host associations of *Melanoconion* mosquitoes are poorly known, and for the majority of species of this diverse group, remain uncharacterized. Among those for which blood meal analysis data are available, a range of host associations have been identified. These data suggest that some *Melanoconion* species (e.g., *Cx. iolambdis*, *Cx. erraticus*) are generalists that feed from a broad range of hosts, including all terrestrial vertebrate classes [17,29,45,46,47]. Others primarily feed from mammals (e.g., *Cx. cedecei*, *Culex paracrybda*) with varying extents of specialization for particular orders [17,30,43,48]. At least one, *Culex peccator*, feeds broadly on ectothermic hosts (reptiles and amphibians), with no particularly strong association with lizards [47,49]. Tempelis and Galindo [48] and Christensen et al. [45] investigated the host use patterns of mosquitoes in Panama while using serological methods, and found that several *Melanoconion* species (*Culex conspirator*, *Culex dunni*, *Culex educator*, *Culex elevator*, *Culex egcymon*) fed primarily from lizards, although not as narrowly as *Cx. atratus* and *Cx. pilosus* in the current study. 

In Florida, and elsewhere in the Neotropics, *Melanoconion* mosquitoes are important vectors of VEE virus [6]. Edman [17] stated that “Even though engorged *Cx. atratus* and [*Culex*] *mulrennani* were not encountered and tested in this investigation, their importance in VEE transmission can be safely eliminated on grounds of distribution and abundance”. Recent work indicates that *Cx. atratus* is a common mosquito in Everglades National Park [25] and occurs further north than previously known [29]. Either *Cx. atratus* was present but undetected by Edman [17] in the Everglades, or its distribution and abundance patterns have since shifted. Our results suggest that host associations, rather than distribution and abundance, exonerate *Cx. atratus* as a potential vector of VEE virus. Similarly, the vector of *Plasmodium floridense*, which is a saurian malaria parasite that infects *A. carolinensis*, *A. sagrei*, and *Sceloporus undulatus* (eastern fence lizard) in Florida [50,51], is unknown but presumed to be *Cx. erraticus* [52]. Although *Cx. erraticus* has been found to feed from reptilian (though not lizard) hosts in the southeastern USA, previous work indicates that it more frequently feeds from endothermic hosts, with blood meal analyses finding 2–7% of tested blood meals derived from reptiles [17,47,49,53]. In comparison, we found that reptiles, particularly *Anolis* lizards, made up 99.5% and 97.6% of all identified *Cx. atratus* and *Cx. pilosus* blood meals, respectively. Given the strong association that we observed between *Anolis* lizards and *Cx. atratus* and *Cx. pilosus*, these mosquitoes should be further investigated as potential vectors of *Plasmodium floridense*.

## 5. Conclusions

In Everglades National Park, Florida, *Cx. atratus* and *Cx. pilosus* were strongly associated with reptilian hosts, particularly native and non-native lizards of the genus *Anolis*. The host associations of *Cx. atratus* were previously unknown, and those of *Cx. pilosus* were unclear. Previous investigations of *Cx. pilosus* found diverging patterns of host-use. Our results, which are based on blood meal analysis from 260 *Cx. atratus* and 248 *Cx. pilosus* from multiple sites in Everglades National Park indicate that, in southern Florida, these mosquito species are specialists of reptilian hosts, particularly *Anolis* lizards.

## Figures and Tables

**Table 1:** Sampling localities in and around Everglades National Park, Florida, United States of America (USA) and number of *Culex atratus* and *Culex pilosus* blood meals collected and analyzed from each site. All sites are located within the national park with the exception of C-111E Canal.

Nearest Landmark	Habitat	Coordinates	*Culex atratus*	*Culex pilosus*
C-111E Canal	Hardwood hammock	25.407291, −80.523353	0	2
Anhinga Trail	Hardwood hammock	25.402178, −80.615617	31	85
Long Pine Key	Pine rocklands	25.399878, −80.659990	27	65
Pa-Hay-Okee	Sawgrass prairie	25.412619, −80.782649	11	15
Mahogany Hammock	Hardwood hammock	25.338870, −80.818099	82	34
Nine-Mile Pond	Mangrove	25.253915, −80.798166	24	21
Snake Bight	Mangrove	25.200773, −80.874253	43	12
Buttonwood Canal	Coastal prairie	25.148854, −80.923344	41	11
Flamingo	Coastal prairie	25.144328, −80.921075	1	3
TOTAL			260	248

**Table 2:** Primer pairs used to amplify vertebrate host DNA from *Culex atratus* and *Culex pilosus* blood meals that were collected in southern Florida, USA.

Primer Name	Gene	Primer Sequence	Amplicon Size (bp)	Reference
VertCOI_7194_F	COI	5′-CGM ATR AAY AAY ATR AGC TTC TGA Y-3′	395	[32]
Mod_RepCOI_R		5′-TTC DGG RTG NCC RAA RAA TCA-3′		
Mod_RepCOI_F	COI	5′-TNT TYT CMA CYA ACC ACA AAG A-3′	244	[32]
VertCOI_7216_R		5′-CAR AAG CTY ATG TTR TTY ATD CG-3′		
Mod_RepCOI_F	COI	5′-TNT TYT CMA CYA ACC ACA AAG A-3′	664	[32]
Mod_RepCOI_R		5′-TTC DGG RTG NCC RAA RAA TCA-3′		
16L1	16s	5′-CTGACCGTGCAAAGGTAGCGTAATCACT-3′	450	[29,30,31]
H3056		5′-CTCCGGTCTGAACTCAGATCACGTAGG-3′		

**Table 3:** Results of blood meal analysis for *Culex atratus* and *Culex pilosus* from southern Florida. For each mosquito species the number (n) of blood meals derived from each host species, and the percentage (%) of identified blood meals attributed to each host are indicated. Mixed blood meals refer to blood meals derived from more than one host animal. No amplification refers to blood meals that did not produce a PCR amplicon, and as a result were not identified.

Host Class	Host Species	*Culex atratus*		*Culex pilosus*	
		n	%	n	%
Reptilia	*Anolis carolinensis*	43	20.6	18	10.7
Reptilia	*Anolis sagrei*	163	78	137	81.5
Reptilia	*Anolis equestris*	0	0	1	0.6
Reptilia	*Coluber constrictor*	0	0	1	0.6
Reptilia	*Nerodia fasciata*	0	0	1	0.6
Reptilia	*Alligator mississippiensis*	2	1	2	1
Reptilia	*Crocodylus acutus*	0	0	3	1.7
Reptilia	*Terrapene carolina*	0	0	1	0.6
Amphibia	*Hyla cinerea*	0	0	1	0.6
Aves	*Cardinalis cardinalis*	0	0	1	0.6
Aves	*Nyctanassa violacea*	0	0	1	0.6
Mammalia	*Homo sapiens*	0	0	1	0.6
	Mixed blood meal	0	0	1	0.6
	No amplification	52		80	
	Total identified	208		168	
	TOTAL	260		248	
